# Current status of cystic echinococcosis in West Africa: a silent zoonotic risk in humans and animals

**DOI:** 10.1186/s41182-025-00825-2

**Published:** 2025-11-20

**Authors:** Shigehiro Enkai, Cornelia Appiah-Kwarteng

**Affiliations:** 1 Asia International Institute of Infectious Disease Control and Department of Pediatrics, Teikyo University School of Medicine, 2-11-1 Kaga, Itabashi-Ku, Tokyo, 173-8605 Japan; 2 School of Veterinary Medicine, College of Basic and Applied Sciences, University of Ghana, Accra, Ghana

## Abstract

Cystic echinococcosis (CE) is a zoonotic disease caused by the larval stages of the cestode *Echinococcus granulosus* sensu lato (s.l.). The spread of CE results in significant economic and health damage to endemic regions. Despite its importance, there is a paucity of information regarding CE in West Africa. However, case reports from West African immigrants and refugees suggest the potential disease risk for humans in the region. Research on the prevalence of CE in livestock is also limited, with the figures showing considerable variation by year and location. Genetic studies of parasite samples in Nigeria, Mali, Mauritania, and among immigrants in Europe have predominantly identified the G6 and G6/7 genotypes of *E. canadensis*, which mainly infect camels. The G1 genotype was also identified in camels in Nigeria and immigrants from West Africa. As the intermediate hosts, camels are the major factor contributing to CE in West Africa. Furthermore, the infection rate in dogs, the definitive host, is 0.5–12.3% in the study area. Notably, the life cycle of the parasite is sustained by stray dogs that interact with animal carcasses and improper slaughterhouse waste disposal. *Echinococcus* is present in humans and animals in West Africa. It is critically important to enhance veterinary training and public health education, as well as maintain surveillance systems, to prevent human CE cases and economic damage in West Africa.

## Background

Cystic echinococcosis (CE) is caused by the larval stage of *Echinococcus granulosus* s.l, one of the most harmful and life-threatening helminths. *E. granulosus* s.l*.* has a global distribution and is found in Africa, North and South America, Australia, and many regions of Eurasia [1, 2]. The main definitive hosts are feral carnivores such as wolves and dogs. The intermediate hosts are wild and domestic ungulates such as sheep, cattle, pigs, and camels. Adult parasites living in the small intestine of their definitive host release their eggs into the host’s feces. Humans are accidentally infected by ingesting the eggs. *E. granulosus* s.l*.* is differentiated into 10 genotypes (G1–G10) based on genomic patterns [3–5]. They are characterized by differences in morphology and host specificity. G1, G2, and G3 genotypes are identified as *E. granulosus* sensu stricto (s.s.), which were isolated from common sheep (G1, G2) and a buffalo strain (G3). The G1 strain, which is responsible for the majority of human CE cases worldwide, is the most prevalent genotype. The G4 and G5 strains correspond to *E. equinus*, commonly found in horses, and *E. ortleppi*, typically associated with cattle. G6, G7, G8, and G10 genotypes are classified within the species *E. canadensis*. The G6 genotype is commonly associated with camels, while the G6/G7 genotype is found in camels and cattle. The G7 and G8 genotypes are typically identified in pigs and cervids, respectively, and the G10 genotype is known as the Fennoscandian cervid strain. It is rare for G8 and G10 genotypes to be reported as human infections. The G9 strain remains unidentified. The G1 strain, known as *E. granulosus* s.s., is the predominant cause of human infections and is extensively found in Eastern Europe, South America, the Middle East, the Tibetan region, and North Africa, where sheep farming is common [6]. The second most prevalent genotype is the G6 strain of *E. canadensis*, which poses public health concerns in the Middle East and North Africa, particularly in areas with significant camel breeding industries [7]. In economically unstable regions, the spread of CE can cause significant financial damage [8]. This review focuses on West Africa and provides a summary of the current status and future outlook of *E. granulosus* s.l infection in humans, livestock (intermediate hosts), and dogs (definitive hosts), based on the limited available literature.

## Method

The primary research and reviews published between 1970 and 2025 were searched via PubMed and Google scholar, using the search terms ‘‘Echinococcus” or ‘‘hydatid” in combination with ‘‘genotype”, ‘‘livestock”, ‘‘camel” or ‘‘human case” in relation to the countries of West Africa. In the papers regarding livestock, the most recent reports were prioritized as much as possible.

## Diagnostic approaches for *E. granulosus* s.l infection

Detection of CE in livestock is usually performed through visual inspection during post-mortem examinations at slaughterhouses [9]. However, distinguishing lesions caused by *E. granulosus* s.l. from those due to other diseases can be challenging, especially when lesions are small, which requires the use of polymerase chain reaction (PCR) tests or histopathological analysis. A definitive diagnosis can be made if protoscoleces are observed inside cysts under a microscope or on pathological examination, although caution is necessary since some animal species may not develop protoscoleces. PCR targets sequences from the 12S rRNA, cox1 and/or nad1 genes to analyze the parasite tissues [10, 11]. A systematic review of genotyping *E. granulosus* s. l. showed that 91.8% of studies sequenced the cox1 gene, whereas 40% sequenced the nad1 gene [12]. For human diagnosis, imaging techniques such as ultrasound and computed tomography (CT) are standard, although serological tests are also sometimes used [13]. Serological diagnosis generally uses Antigen 5, Antigen B (AgB), EgAgB8/1, and EgAgB8/2, proteins that constitute the cyst, as antigens [14, 15]. ELISA is used as a screening tool, with immunoblotting occasionally added to enhance diagnostic accuracy. When examining the parasite in the feces of canid definitive hosts, copro-ELISA or PCR including real-time PCR is used to detect specific antigens or DNA from adult worms, often in combination with egg detection methods, such as flotation techniques [9, 16, 17].

## The current status of *E. granulosus* in West Africa

### Mauritania

Valuable research on *E. granulosus* s.l*.* was conducted in 2010, which has important implications for public health in Nouakchott, the capital city. Autopsies were performed on stray dogs euthanized to control rabies, and the presence of the adult stage of the parasite was investigated [18]. Of 121 dogs tested, 14% were found to be infected with *E. granulosus* s.l.. Meanwhile, in humans, 24 surgical procedures for CE removal were documented at Nouakchott Hospital within one year of the study [18]. The author estimated that the incidence rate of this condition is 1.2 cases per 100,000 individuals in Mauritania [18]. A 2002 report indicated that the camel strain is actually infectious to humans and circulates between intermediate hosts including camels and cattle. It is suggested that preventive measures at slaughter could reduce the risk of infection in humans [19]. In livestock, CE was detected in 30.1% of dromedary carcasses, 5.5% of cattle, and 6.5% of sheep examined in 2010 [18]. Comparatively, data from 1984 showed a prevalence of 54% in camels, 0.85% in cattle, 2.1% in sheep, and 2.04% in goats [18, 20]. These figures indicate the significant role of camels as intermediate hosts for *E. granulosus* s.l.. Moreover, the G6 and G6/G7 strain cluster, known as the camel/pig strain, was identified in Mauritanian camels in 2007 [21, 22].

### Mali

In 1987, one of 11 camels at a slaughterhouse in northern Mali was found to have CE [23]. Between 2010 and 2011, researchers analyzed fecal and fur samples from dogs in Bamako, Mali’s capital city. Of 193 dogs tested, one dog (approximately 0.5%) was found to carry the G6 genotype of *E. canadensis* [24]. In addition, Yéna et al*.* [25] documented eleven human CE cases in Bamako through a retrospective study covering the period from 1960 to 2000. All patients were diagnosed with pulmonary hydatid disease and underwent surgical removal of the cysts [25].

### Senegal

In 1971, 1973, and 2019, three cases of human CE were reported, respectively [26–28]. There is no scientific research regarding the presence of *E. granulosus* s.l*.* in dogs and domestic livestock in Senegal.

### Cape Verde

The Cape Verde archipelago, an island nation, is situated 600 km off the coast of Senegal in the North Atlantic Ocean. Researchers conducted coprological and genetic analyses of environmental dog feces and tissue samples from domestic animals in slaughterhouses [29]. Of 369 dog fecal samples, 8 samples (approximately 2.2%) tested positive for *E. granulosus* s.l.. Further, 17 of 40 samples (42.5%) from pigs and cattle were found to contain cysts of the parasite. Genetic analysis identified the G7 genotype in all samples collected from intermediate hosts. In dog fecal samples with echinococcus eggs, one was identified as G6/G7 and the remaining seven as the G7 genotype. No cases of human infection have been reported in Cape Verde.

### Côte d’Ivoire

During a post-mortem inspection in 2019 at a slaughterhouse in Abidjan, the capital city, a total of 2524 pigs were examined [30]. Of these, 101 cases were suspected as CE. Following cytopathological and histopathological testing, 61 were identified as CE. This study did not perform genotyping. Additionally, a para-clinical analysis of isolated bovine organs was conducted at a slaughterhouse located in the southern part of Abidjan from 2008 to 2015 to detect CE [31]. This retrospective study was based on data from the annual surveillance reports of all cattle inspected at the facility. Over the eight-year period, the prevalence of bovine CE in isolated organs was 1.6%, and all positive cysts were identified in the kidneys. Historical human cases include three that were reported in 1978; however, the details are unknown [32]. More recently, in 2023, two men residing in Bouaké, located in the northern region, were diagnosed with CE in the liver [33, 34].

### Burkina Faso

The Ministries of Health and Animal Resources conducted surveys to determine the prevalence of CE in cattle and pigs slaughtered in the years 1996 and 1997 [35]. CE was detected in 10 of the 135,822 cattle inspected, representing a 0.007% occurrence rate. On the other hand, no cases of infection were found among the 117,026 pigs examined. Data concerning camels and dogs were not included in the report. No cases of CE in humans have been reported in Burkina Faso [36].

### Ghana

There have been no reported cases of CE in humans in Ghana, nor have there been investigations into domestic animals and dogs. However, three instances of CE in Ghanaian immigrants have been documented in the USA and Europe [37, 38].

### Nigeria

Nigeria has conducted the most extensive research on CE in West Africa. Reports during the past 30 years showed the high prevalence of CE in domestic animals in the northern region of Nigeria. In particular, camels have the highest incidence of CE among domestic animals [11, 39]. In Kano State, the rate of CE found in animals slaughtered for food was 14.7% in cattle, 11.4% in sheep, 26.5% in goats, and 55.5% in camels in 1980 [40]. In 2018, a study focusing on CE was carried out, which involved the visual inspection of 404 camels slaughtered at the Maiduguri slaughterhouse. The findings revealed an overall prevalence rate of 1.73%, with 7 cases identified [41]. A study in 2019 revealed the infection rates for *E. granulosus* s.l*.* among different livestock [11]: 19.49% in camels (23 of 118), 0.47% in cattle (4 of 856), 0.31% in goats (1 of 318), and 0% in sheep (0 of 300). The genotype of all cysts obtained from this study was G6/G7, *i.e.*, *E. canadensis*, the camel strain. In Maiduguri, 304 camels and 256 cattle were visually inspected for CE at the slaughterhouse in 2022. The results showed that 14.1% of camels and 1.6% of cattle had CE. Furthermore, blood samples were taken from each animal for serological testing. These tests revealed a seroprevalence of 52.6% in camels and 35.5% in cattle [42]. Based on these studies, it is estimated that the recent infection rate of CE in livestock in northern Nigeria is between 14 and 19% in camels and 0.47% and 1.6% in cattle. Regarding dogs, in a 1979 study conducted in Kano State, *E. granulosus* s.l*.* eggs were detected in nine (6.21%) of the 145 stray dogs examined [40]. A more recent 2022 investigation at an animal hospital in the northeast evaluated stool samples from 470 dogs and found that 12.3% were positive for *E. granulosus* s.l*.* eggs [43]. However, consolidated surveillance information regarding the infection status in humans is lacking.

There have been multiple surveys in the southern region of Nigeria as well. Research conducted in the Niger Delta area in 1985 showed infection rates of 56% in pigs, 32% in cattle, 42% in goats, and 24% in sheep [44]. Regarding dogs, in Oyo, Ogun, Osun, Ekiti, Ondo, and Lagos States, the sera of 273 dogs were tested for the presence of echinococcus antigen using direct ELISA in 2014, and the total prevalence was 12.4% [45]. However, it should be noted that concurrent fecal examination for parasite eggs was not performed. In 2021, stool samples from dogs that had visited veterinary clinics in Lagos State were examined using multiplex PCR to detect parasite eggs [46]. Of 217 dogs, *E. granulosus* s.l*.* was found in 12 (5.5% infection rate). In Ibadan, near Lagos State, indirect ELISA tests were conducted to evaluate antibodies in 185 dogs that had visited veterinary clinics in 2023. The results indicated that 33.5% of the dogs were seropositive for CE infection [47]. Interestingly, in a 2022 investigation, soil, fecal, and water samples from areas inhabited by stray dogs in Ibadan were tested for echinococcus eggs using microscopic observation and multiplex PCR. The analysis revealed the presence of *E. granulosus* s.l*.* eggs in 8.0% of soil (n = 200), 24.0% of fecal (n = 200), and 2.0% of water samples (n = 50) [48]. As for human cases, there have been some reports of CE in individuals from Anambra, Osun, and Ibadan in 1989, 2007 and 2017, respectively [49–51]. A case of cerebral echinococcosis was documented in rural southwestern Nigeria in 2024 [52]. However, there are no survey data available on the genetic classification of samples in the southern region. Therefore, the predominant genotype(s) in the south remains unknown.

### Niger

In 1991, two hospitals reported a total of 32 cases of CE in humans [53]. Research on slaughtered animals showed that CE was found in camels, but not in sheep; however, the specific details remain unclear [53]. A Nigerien refugee in Italy was found to have the G6 genotype of *E. canadensis*, which is typically associated with camels [54].

### Others

There have been no domestic surveys conducted in Gambia, Guinea-Bissau, Guinea, Liberia, Sierra Leone, Togo, or Benin concerning human or animal cases of CE.

## Refugees or immigrants from West Africa

**Fig. 1:**
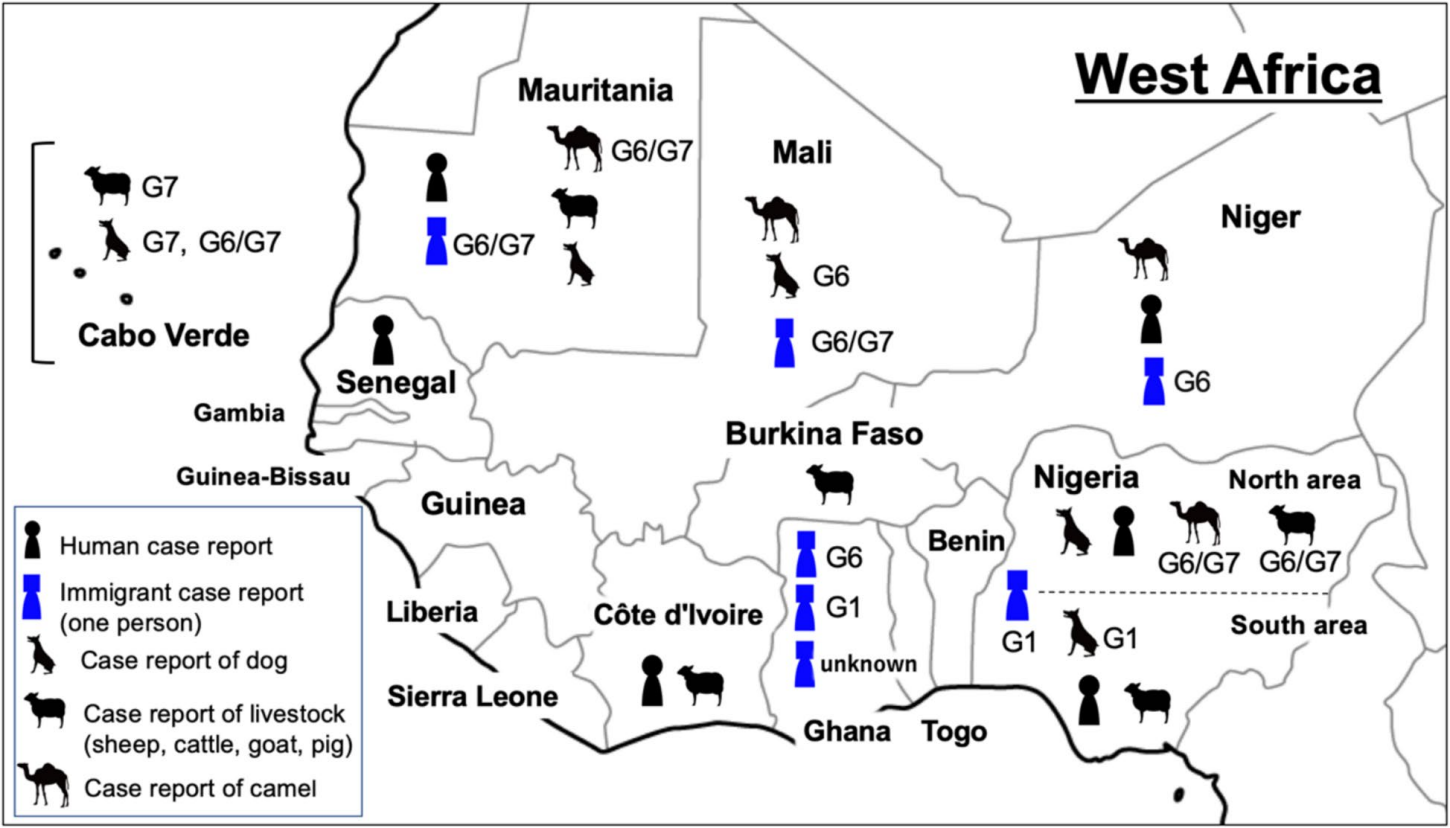
Hosts and genotypes of *E. granulosus* s.l*.* reported in West Africa from 1980 to 2025. Ongoing comprehensive reporting for the parasite is lacking. While G6 and G6/7 genotypes, the camel types, have been reported in many cases, the G1 genotype was detected in migrants from Ghana and Nigeria

Cases of immigrants and refugees from West Africa infected with CE have been reported (Fig. 1): one each from Niger [54], Nigeria, Mauritania, and Mali [38], and three from Ghana [37, 38]. Genotypic analysis revealed two cases with the G6 genotype from Ghana and Niger, and two cases with the G6/G7 genotype from Mauritania and Mali (Table 1). Additionally, there were two cases of the G1 genotype from Ghana and Nigeria. One case had no genotype analysis. All cases were diagnosed after relocating to Europe or the USA. Although detailed information is typically lacking in these cases, the case of the Ghanaian diagnosed in the USA is noteworthy. The individual had resided in Accra and had not visited any country besides the USA. This suggests that CE could be a potential health risk even in the southern regions of Ghana. In West Africa, there are few areas where populations can receive hospital diagnostic and treatment services. Thus, many cases might be treated as tumors or natural deaths, and even if diagnosed, they may not be reported in case studies.

**Table 1:**
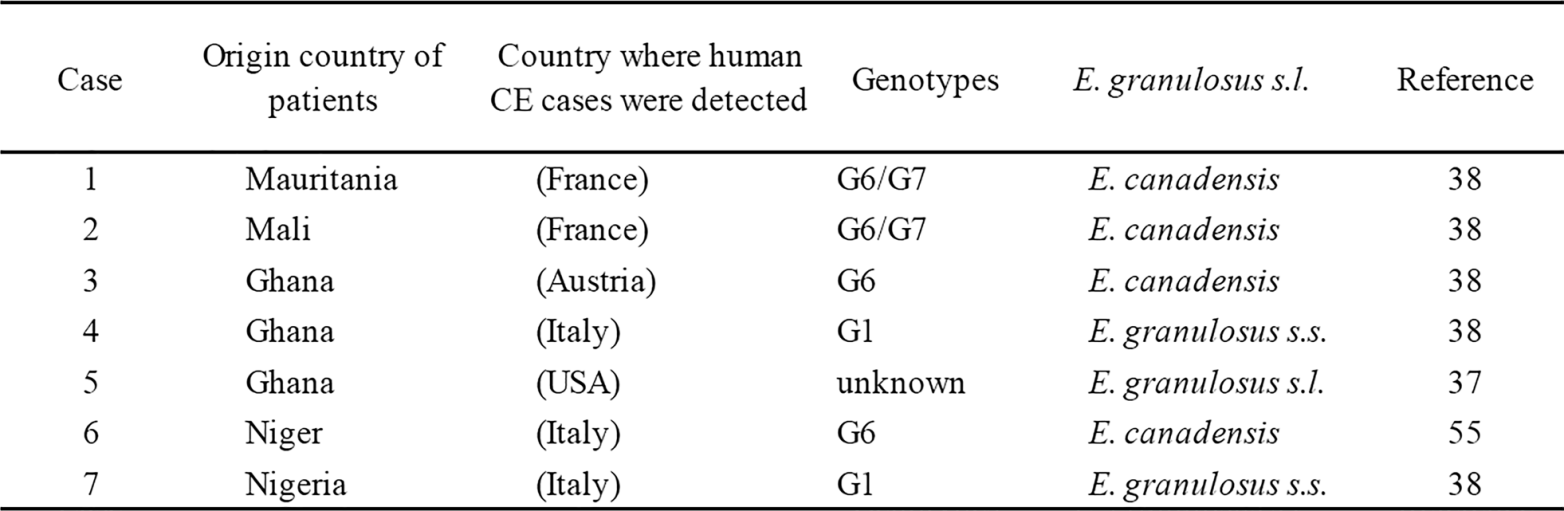
Imported cases of CE to other area from West Africa in past reports

### Dogs

**Fig. 2:**
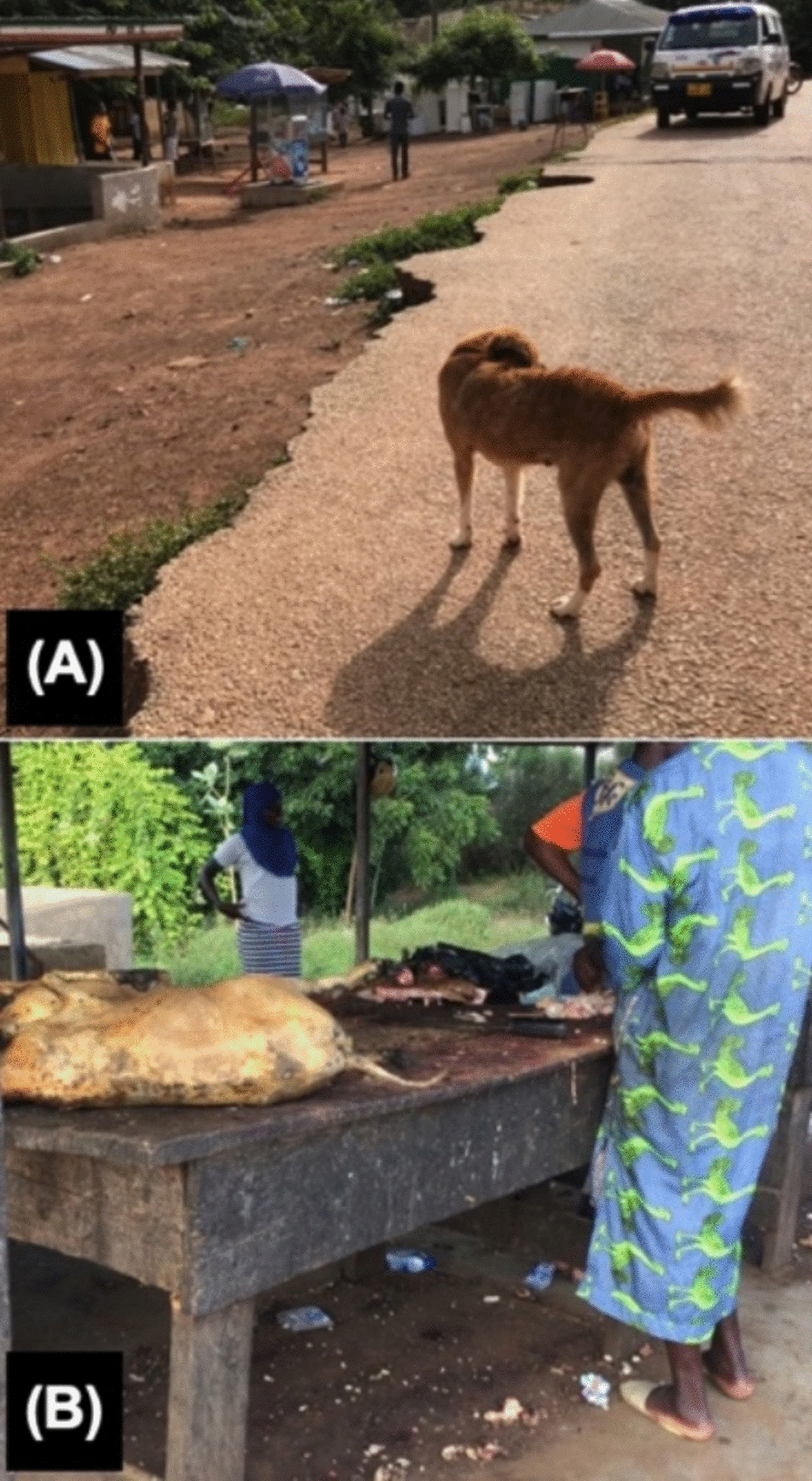
**A** Free-range dogs in a village in northern Ghana. The management of stray dogs is a significant issue in Africa. **B** In a local meat market, contact between discarded organs after slaughter and stray dogs increases the risk of echinococcosis infection. Meat management is a significant challenge for the livestock industry. The photo is from northern Ghana

Dogs play a crucial role as definitive hosts for *E. granulosus* s.l. The infection rate in dogs is 14% in Mauritania, 0.5% in Mali, 2.2% in Cape Verde, and 5.5%–12.3% in Nigeria [18, 24, 29, 40, 43, 46, 47]. They become infected by consuming the liver and other organs of animals improperly discarded from homes or slaughterhouses. As long as there is contact between dogs and discarded offal, the cycle of infection will continue (Fig. 2). Dogs in Africa are primarily kept to protect livestock or households. However, due to economic constraints, the majority of dog owners lack the financial resources to fence their properties and properly care for their dogs, resulting in dogs frequently roaming the streets [55]. In addition, cultural factors contribute significantly to the low number of individuals willing to assume responsibility for dogs. These factors include the perceived unpleasant odor of dogs, their unhygienic behaviors, and religious or superstitious beliefs that discourage ownership of dogs [56]. Consequently, the management of free-roaming dogs remains a significant challenge, compounded by both socioeconomic limitations and cultural perceptions.

## CE genotypes found in West Africa

Previous reports have indicated that the genotypes G6, G6/G7, and G1 are present in West Africa and vary according to the population of intermediate hosts in the region (Fig. 1). Cases of CE in refugees or immigrants might reflect the current state of CE prevalence in this area. *E. canadensis* (G6, G6/G7) is the predominant cause of CE in both humans and animals across West Africa. It has been reported that *E. canadensis* (G6/7) accounts for 97.4% of the genotypes in West Africa, although the availability of genotype information is limited [57]. Pigs can also serve as an intermediate host for the G6/G7 genotype. However, the majority of G6/G7 samples have been isolated from camels in West Africa. Therefore, it appears that pigs play a less prominent role in the epidemiology of the G6/G7 genotype in this region than camels. Based on reports to date, the genotypes G6 and G6/G7 are the most prevalent, and camels play a significant role as hosts in West Africa. On the other hand, the first instance of the G1 genotype was identified in a Nigerian camel in 2019 [58]. Typically, camels are the primary intermediate hosts for the G6 and G6/G7 genotypes. However, cases of camels infected with the G1 genotype, which is usually associated with sheep, have been reported in endemic regions such as the Middle East and North Africa [59, 60]. These observations suggest that the shared livestock environment of sheep and camel farming could potentially lead to a rise in G1 infections among them. The genotypes *E. granulosus* s.s*.* G1 and *E. canadensis* G6/7 account for 88.4% and 7.3% of human CE cases worldwide, respectively [6]. The G1 genotype is responsible for the majority of human CE cases. Consequently, the spread of the G1 genotype through camels may be associated with an increased risk of human CE infection. In recent years, there has been a shift towards camel farming from cattle, since camels are better able to tolerate heat and drought with climate change [61, 62]. It may be necessary to exercise greater caution in the management of camels, which serve as intermediate hosts for CE, in the future. CE-infected pigs, cattle, sheep, and goats are found in the southern regions of Côte d’Ivoire and Nigeria. Notably, the genetic survey was limited to the northern regions of West Africa and was not conducted in the southern areas.

## Conclusion

Echinococcus is present in humans and animals in West Africa. The prevalence rate of *E. granulosus* s.l. across West Africa shows significant variation depending on the survey year, region, and intermediate hosts, indicating the need for fixed-point surveys. The predominant endemic genotypes are G6 and G6/7, detected in camels. In recent years, G1 types have also been detected in camels, dogs, and humans. There is very little information available about echinococcosis in West Africa. The establishment of surveillance systems and strengthening of diagnostic capacity for genotyping of *E*. *granulosus* in the region are needed for effective control to safeguard animal and public health.

## Data Availability

No datasets were generated or analysed during the current study.
